# LS-VIT: Vision Transformer for action recognition based on long and short-term temporal difference

**DOI:** 10.3389/fnbot.2024.1457843

**Published:** 2024-10-31

**Authors:** Dong Chen, Peisong Wu, Mingdong Chen, Mengtao Wu, Tao Zhang, Chuanqi Li

**Affiliations:** ^1^College of Physics and Electronic Engineering, Nanning Normal University, Nanning, China; ^2^College of Computer Science and Engineering, Guangxi Normal University, Guilin, China; ^3^Guangxi Key Laboratory of Functional Information Materials and Intelligent Information Processing, Nanning, China

**Keywords:** action recognition, motion extraction, temporal crossing fusion, Vision Transformer, deep learning

## Abstract

Over the past few years, a growing number of researchers have dedicated their efforts to focusing on temporal modeling. The advent of transformer-based methods has notably advanced the field of 2D image-based vision tasks. However, with respect to 3D video tasks such as action recognition, applying temporal transformations directly to video data significantly increases both computational and memory demands. This surge in resource consumption is due to the multiplication of data patches and the added complexity of self-aware computations. Accordingly, building efficient and precise 3D self-attentive models for video content represents as a major challenge for transformers. In our research, we introduce an Long and Short-term Temporal Difference Vision Transformer (LS-VIT). This method incorporates short-term motion details into images by weighting the difference across several consecutive frames, thereby equipping the original image with the ability to model short-term motions. Concurrently, we integrate a module designed to understand long-term motion details. This module enhances the model's capacity for long-term motion modeling by directly integrating temporal differences from various segments via motion excitation. Our thorough analysis confirms that the LS-VIT achieves high recognition accuracy across multiple benchmarks (e.g., UCF101, HMDB51, Kinetics-400). These research results indicate that LS-VIT has the potential for further optimization, which can improve real-time performance and action prediction capabilities.

## 1 Introduction

The rise in the popularity of short video content has led to an increase in the public willingness to share snippets of their daily lives on various social media platforms. Therefore, the internet is flooded with videos every day, turning these visual snippets into a treasure trove of information ripe for video analysis. The information embedded in these videos, especially the dynamic actions they capture, is closely correlated with temporal aspects–consider the difference between closing and opening a door. Overlooking the temporal dimension can lead to misinterpretation of these actions as identical, highlighting the importance of accurately modeling time in video analysis. The prevailing method for understanding videos efficiently involves mapping out temporal information on a graph, which is then analyzed utilizing 2D Convolutional Neural Networks (CNNs) (Wang et al., 2016; Zhou et al., 2018; Karpathy et al., 2014; Simonyan and Zisserman, 2014). However, this approach encounters a significant hurdle with respect to temporal modeling. Specifically, 2D CNNs, when applied to single frames, does not suffice in capturing the essence of temporal information, presenting a major challenge in recognizing video actions over time. In response to this challenge, the field has witnessed significant advancements in video action recognition, largely fueled by the advent of 3D Convolutional Neural Networks (3D CNNs) and their factorized variants (Carreira and Zisserman, 2017; Feichtenhofer et al., 2018; Lin et al., 2019; Qiu et al., 2017). These networks are adept at learning both spatial and temporal patterns, albeit at the cost of higher computational demands (Tran et al., 2015; Chen et al., 2018). An alternative strategy has been introduced, focusing on the implicit learning of motion characteristics from static images, either through factorized versions or by leveraging temporal convolutional features (Xie et al., 2018; Tran et al., 2017; Qiu et al., 2017). Thus, crafting an effective temporal module for 2D CNNs that features a robust capability for motion detection while maintaining low computational requirements remains a challenge.

The burgeoning success of Transformer-based methodologies in image (Dosovitskiy et al., 2021; Liu et al., 2021; Yuan et al., 2021; Han et al., 2021; Touvron et al., 2020; Chen et al., 2021) processing tasks has spurred researchers to extend this success to video processing challenges (Bertasius et al., 2021; Arnab et al., 2021; Liu Z. et al., 2022). In particular, video data is segmented into 3D patches, which are then analyzed utilizing a combination of Self-Attention (SA) mechanisms and Feed-Forward Networks (FFN) for the extraction of spatio-temporal features. The introduction of the temporal dimension in video data significantly amplifies the quantity of patches, thereby causing a surge in both computational and memory requirements. This surge is attributed to the quadratic increase in the complexity of computations for the multi-head SA, a cornerstone of the Transformer architecture. Efforts to mitigate the computational demands of spatio-temporal multi-head SA have traditionally concentrated on partitioning the computation across spatial and temporal dimensions for separate processing (Bertasius et al., 2021; Arnab et al., 2021). For instance, Timesformer (Bertasius et al., 2021) implements an approach where an initial spatial-only SA is applied in the Transformer encoder, followed by a temporal-only SA. Similarly, ViViT (Arnab et al., 2021) incorporates additional temporal-only Transformer encoders after the initial spatial-only encoder phase. Despite these advancements, such factorization methods invariably introduce extra parameters and increase computational load for temporal SA, especially when compared to spatial-only Transformer networks. This raises a critical question: Is it possible to imbue 2D Transformers with the capability to model temporal SA effectively, thus bridging the gap between image-based and video-based tasks without significantly increasing the parameter count and computational burden, as 2D CNNs learn motion features through temporal convolution.

To address the challenges presented, our approach is anchored in the theoretical foundation of short-term and long-term temporal modeling as introduced by TDN (Wang et al., 2021). We introduce a network termed Long and Short-term Temporal Difference Vision Transformer (LS-VIT) that adeptly captures spatio-temporal Self-Attention (SA) features. LS-VIT is inspired by the Vision Transformer (ViT) (Dosovitskiy et al., 2021) image model, yet innovatively incorporates the Long-term Motion Information Module (LMIM) and the Short-term Motion Information Frame (SMIF). These modules are uniquely crafted yet serve complementary functions in harvesting temporal information over varying durations, thereby equipping ViT with enhanced spatio-temporal modeling capabilities. In addressing short-term temporal dynamics, we adopt TSN's approach of sparse temporal sampling, leveraging RGB differencing for short-term temporal depiction. This is optimized by the Temporal Difference Inhibition (TDI) method, which mitigates noise disruptions from image differencing, integrating this optimized data back into the original image. This process enriches a single frame with the motion details of adjacent frames, offering the information of movement. Recognizing motion involves a nuanced understanding of both short-term and long-term temporal information. The challenge intensifies when considering the effect of motion subject displacement on action recognition within sparsely sampled images. A significant hurdle is conveying motion information across widely separated frames. LMIM addresses this by streamlining feature channels and applying temporal differencing, thereby enhancing its capability to process long-term temporal information. In addition, both LMIM and SMIF employ bidirectional differencing, a choice aimed at capturing motion-inspired cross-segment variations.

Our LS-VIT introduces a straightforward yet adaptable approach for simulating video motion, facilitating the integration of image task-based Visual Transformer (VIT) methods into video analytics. To comprehensively demonstrated LS-VIT's efficiency, we integrated the Long-term Motion Information Module (LMIM) and Short-term Motion Information Frame (SMIF) in the VIT framework and carried out evaluations utilizing two benchmark datasets, UCF101, HMDB51, and Kinetics-400. The results of these experiments emphasize the robust performance of our LS-VIT across both datasets. Moreover, we undertook a thorough ablation study to further confirm the utility of LMIM and SMIF. Overall, our primary contributions are encapsulated within three key areas:

1. We propose the network of Long and Short-term Temporal Difference Vision Transformer (LS-VIT), a strategy that facilitates effective spatial-temporal Self-Attention (SA) modeling within a 2D Transformers architecture. Our approach is not merely offering an efficient image representation; it also involves design of the module's structure. Through ablation studies on LS-VIT, we collected valuable insights that could steer future studies into temporal difference modeling.

2. We develop the Temporal Difference Inhibition (TDI) method for the Short-term Motion Information Frame (SMIF), which enhances the integration of short-term motion details into the image. It achieves this by reducing the noise disruptions arising from the temporal variances among the omitted segments, thus capturing short-term temporal elements more effectively.

3. We also introduce the Long-term Motion Information Module (LMIM), a module that melds with existing 2D Transformers. Its distinctive feature lies in its capacity to encapsulate the long-term temporal dynamics of video activities without necessitating extra parameters or increasing computational demands.

## 2 Related work

Action recognition represents a fundamental challenge remained to be resolved in vision, prompting the development of numerous deep learning methodologies (Tran et al., 2015; Feichtenhofer et al., 2016b; Rohrbach et al., 2015; Yang et al., 2020; Piergiovanni and Ryoo, 2018; Feichtenhofer et al., 2016a; Martínez et al., 2019; Zheng et al., 2020; Zhao et al., 2018; Li et al., 2020; Qiu et al., 2017). Predominantly, these strategies are divided into two categories based on their underlying network architectures: those based on Convolutional Neural Networks (CNN) and those utilizing Transformers.

### 2.1 CNN-based methods

CNN-based methods represent a cornerstone in action recognition, employing either 3D convolution (Tran et al., 2015; Carreira and Zisserman, 2017; Feichtenhofer et al., 2018) or integrating 2D-CNN (Wang et al., 2019; Lin et al., 2019; Qiu et al., 2017) with temporal modeling to establish robust backbone networks. For instance, Tran et al. introduced C3D (Tran et al., 2015), a 3D CNN derivative of the VGG model, designed to extract spatio-temporal features from video sequences by applying 3D convolution to the VGG framework. Similarly, I3D (Carreira and Zisserman, 2017) extends the concept by transforming the 2D convolutional filters of the Inception V1 model into 3D, enhancing its capacity to analyze video data over time. Another innovative approach, Slowfast (Feichtenhofer et al., 2018), operates through dual 3D-CNN branches that analyze video at varying frame rates–one targeting high and the other low frame rates–to optimize video analysis accuracy. Considering the significant computational demands of 3D-CNN, there appears to be a growing trend toward enriching 2D-CNN with temporal components. For instance, P3D (Qiu et al., 2017) innovatively separates 3D convolution into a combination of 1D temporal and 2D spatial convolutions, progressively incorporating 3D convolution layers. This strategy enables the effective capture of spatio-temporal details in videos, thereby enhancing action recognition capabilities. TSM (Lin et al., 2019) introduces a novel approach by employing a module that facilitates the shifting of sub-channels to the left or right, offering an alternative to the traditional fixed 1D temporal convolution through group weighting. In addition, TEA (Li et al., 2020) integrates a temporal excitation mechanism with an information collecting module to more effectively seize motion details and expand the temporal field of perception. Similarly, TEINet (Liu et al., 2020) merges temporal excitation with inter-frame interaction mechanisms, utilizing a motion enhancement module and in-depth 1D convolution for superior temporal modeling. Nonetheless, despite their advancements, CNN-based approaches face challenges in effectively modeling long-range dependencies in or across video frames, which can hamper their overall performance in action recognition.

### 2.2 Transformers-based methods

Recently, the adaptation of Transformers for 2D vision tasks (Dosovitskiy et al., 2021; Liu et al., 2021; Yuan et al., 2021; Han et al., 2021; Touvron et al., 2020; Chen et al., 2021) has witness a significant surge, especially in video action recognition (Bertasius et al., 2021; Arnab et al., 2021; Liu Z. et al., 2022; Zhang H. et al., 2021). This approach differs significantly from the 3D convolution methods traditionally utilized in CNNs or their factorized variants. Transformers primarily handle the spatio-temporal correlations in videos through a method referred to as spatio-temporal self-attention (SA) modeling. This allows for the computation of all SAs in a block-wise manner. However, it is important to note that this method is both computationally intensive and prone to having too many parameters, similar to the challenges faced with 3D CNNs. In response to these challenges, several studies have introduced methods to lessen the computational load of joint spatio-temporal SA modeling. For instance, Timesformer (Bertasius et al., 2021) introduces a method that segregates the video into individual frame-level segments, applying separate temporal and spatial SA, followed by a temporal SA after each spatial one. ViViT (Arnab et al., 2021) takes a step further in enhancing the capture of temporal dynamics and relationships in videos by layering temporal Transformer encoders atop the spatial encoder outputs. Video Swin Transformer (Liu Z. et al., 2022), on the other hand, aims at reducing both computational complexity and memory usage by segmenting the local window across both spatial and temporal dimensions for self-attention. TPS (Xiang et al., 2022) distinguishes itself by incorporating a block-transfer and channel-transfer module, aiming to optimize the Transformer's ability to model temporal aspects.

Currently, the predominant method for modeling time series in videos utilizes 2D and 3D CNNs. TSM (Lin et al., 2019) was among the first to suggest temporal modeling between video frames to effectively understand motion features. TRN (Zhou et al., 2018) builds upon this by integrating multiscale features across the temporal dimension to bolster temporal inference. STM (Jiang et al., 2019) introduces a novel block, deviating from the traditional residual block, to better represent spatio-temporal and motion features. Both TEA (Li et al., 2020) and TEINet's (Liu et al., 2020) innovations lie in utilizing temporal difference operations to architect their network models. TDN (Wang et al., 2021) adopts a multi-scale temporal difference modeling strategy to capture motion details comprehensively, facilitating end-to-end action recognition. TPS (Xiang et al., 2022), building upon TSM's foundation, offers block transfer as a complementary enhancement. The effectiveness of temporal modules in 2D CNNs inspired the development of LS-VIT, aimed at enhancing spatial Transformers with the capacity for spatio-temporal feature learning without the necessity for additional parameters. In addition, SMIF and LMIM introduce methods for generating images with short-term motion information features, the former through temporal differencing and the latter by reducing channel feature numbers, addressing the issue of excessive motion information variance between successive frames.

## 3 Methodology

In this section, we evaluate the architecture of the Long and Short-term Temporal Difference Vision Transformer (LS-VIT), which converts spatial Transformers to spatio-temporal Transformers through the integration of the Long-term Motion Information Module (LMIM) and the Short-term Motion Information Frame (SMIF). We will further describe both the LMIM and SMIF approaches specifically.

### 3.1 Overview

**Figure 1:**
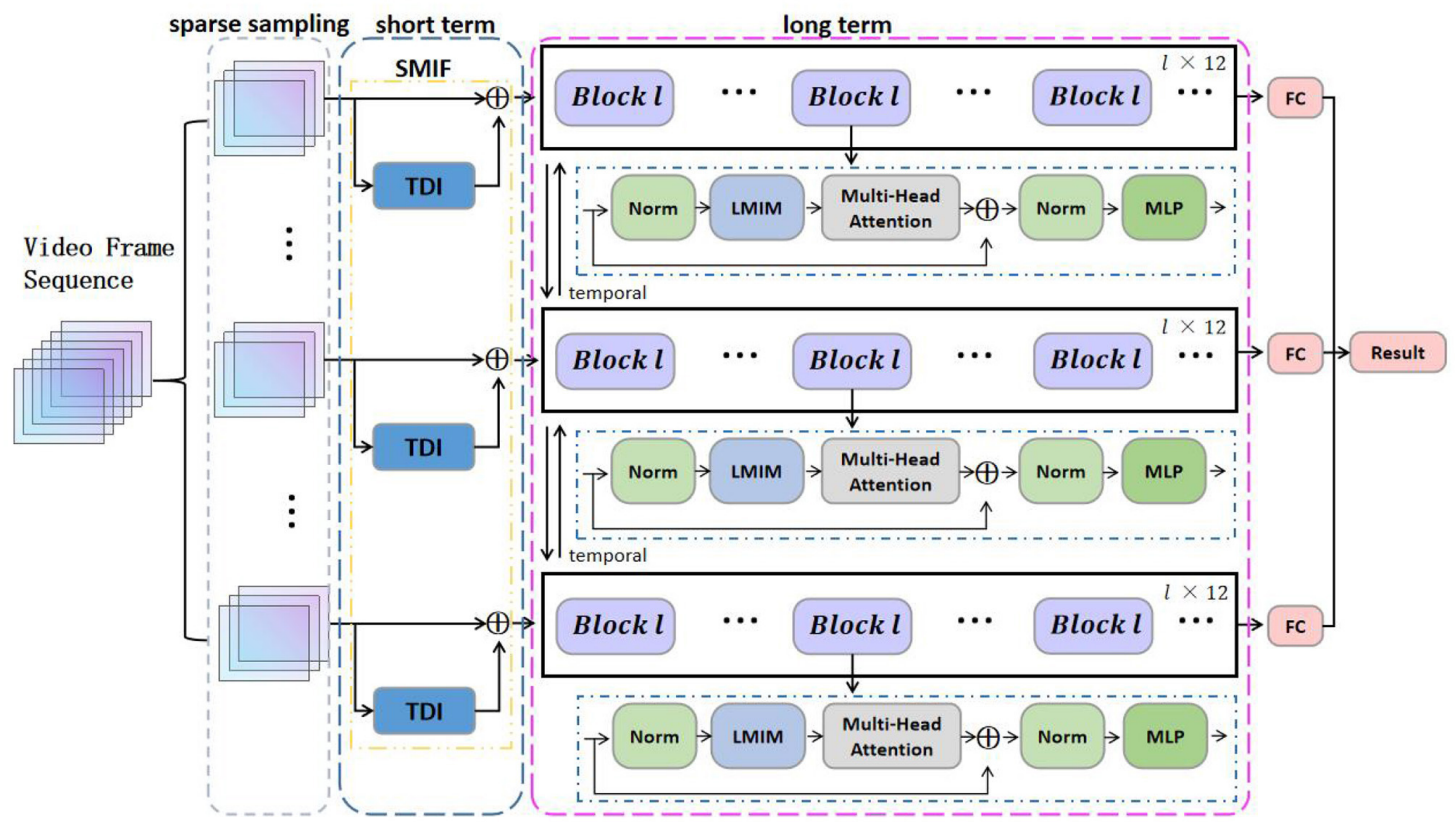
Summary the overall framework of long and short-term motion differences. The SMIF effectively transforms multiple sparsely sampled motion segments into a series of keyframes. These keyframes are then fed into a Vision Transformer (VIT) model integrated with a Long-term Motion Information Module (LMIM). The VIT shares the same parameters across all segments.

Illustrated in Figure 1, our Long and Short-term Temporal Difference Vision Transformer (LS-VIT) is proposed for video-level action learning and leverages the full spectrum of video data. Our primary contribution lies in the application of temporal difference operators at both the network's front end and internally within the Vision Transformer (VIT) framework. In constructing the LS-VIT, we prioritize the integration of short-term motion data right at the input phase to establish the Short-term Motion Information Frame (SMIF). Concurrently, we incorporate a pluggable Long-term Motion Information Module (LMIM) incorporated into the architecture via residual connections, selectively reducing channel information to mitigate issues of motion misalignment that arise with long-term motion.

Videos vary in length, and we address this by dividing a given video, *V*, into *T* non-overlapping segments. From each segment, we extract frames, referred to as *X*, characterized by the shape of [*B, T, C, H, W*]. where *B* represents the batch size or the number of videos processed simultaneously, *T* denotes the sequence length of the frames, *C* stands for the number of feature channels, and *H* and *W* signify the frame's height and width, respectively. This division results in a total of *T* frames *I* = [*I*_1_, …, *I*_*T*_], where the shape of *I* is [*C, H, W*]. The SMIF is designed to enrich the image with localized motion details, thereby enhancing its descriptive capability:


(1) $$\begin{array}{l} SMIF:\hat{I_{i}}=I_{i}+D\left(I_{i}\right) \end{array}$$

Where $\hat{I_{i}}$ denotes the image enhanced with short-term motion details, extracted from analyzing motion between adjacent frames of *I*_*i*_, and *D* represents the differential image highlighting frame changes. *F* denotes the frame-level attributes extracted by feeding the 2D Transformer with these frames, where *F* = [*F*_1_, …, *F*_*T*_]. The LMIM mainly utilizes the temporal structure across frames to enhance the representation of frame-level features:


(2) $$\begin{array}{l} LMIM:\hat{F_{i}}=F_{i}+F_{i}⊙\text{L}\left(F_{i}\right) \end{array}$$

Where, *F*_*i*_ represents the features input to the encoder, and **L** denotes the LMIM module, which is inserted into the Self-Attention (SA) module of an existing 2D Transformer block. This module aggregates information from other temporal frames, transforming the spatial features *F*_*i*_ into spatiotemporal features $\hat{F_{i}}$. This approach enables the model to comprehend the temporal structure within extended motion sequences by layering multiple LMIMs. The detailed methodology will be described in the following subsection.

### 3.2 Short-term motion information

In the analysis of action videos, we have observed that the data from adjacent frames are highly correlated, selecting a single frame from each video segment captures only the spatial aspects of the action will inevitably overlook crucial temporal information. Therefore, we propose a Short-term Motion Information Frame (SMIF) method, which produces an RGB image for each frame containing motion information that integrates the visual appearance and motion details of the neighboring frames, characterizing the video information in terms of images. Through this approach, a more comprehensive and profound understanding and analysis of video action information is achieved.

**Figure 2:**
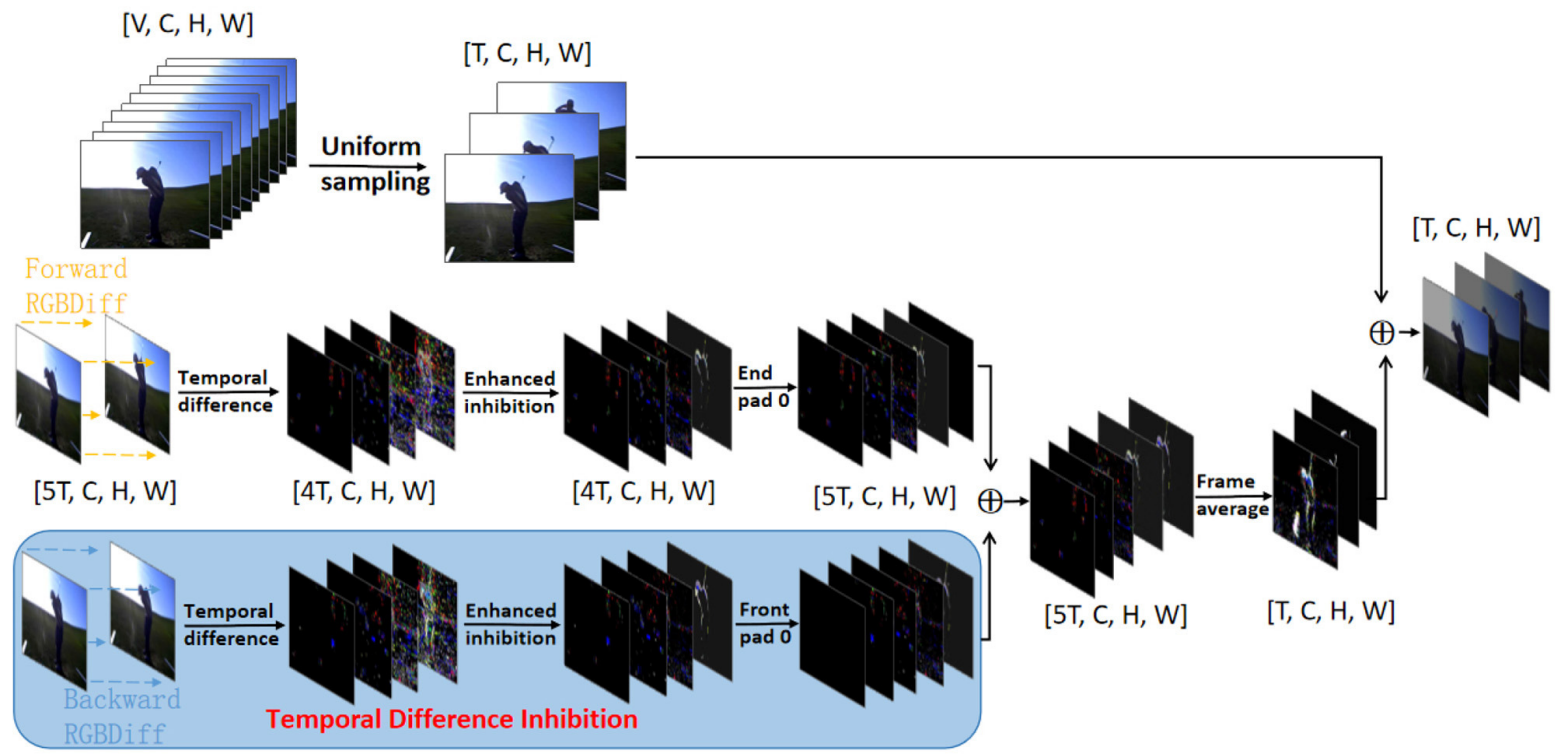
Overview of the proposed Short-term Motion Information Frame. The resulting short-term frame difference image is combined with the original image by differencing the original image after doing time difference suppression on it. ⊕ denotes the element-wise sum.

Specifically, our SMIF approach is specifically designed to enhance the network's input by integrating the temporal differences across frames, thereby enabling the derived single-frame RGB images to encapsulate local movement. As depicted in Figure 2, for each sampled frame *I*_*i*_, we extract the two adjacent frames immediately before and after it, in a localized window centered on *I*_*i*_ to make a short-term image set *S*_*i*_ = [*I*_*i*−2_, *I*_*i*−1_, *I*_*i*_, *I*_*i*+1_, *I*_*i*+2_]. We then calculate the differences between these consecutive frames in pairs to identify short-term temporal difference sequence *D*_*i*_:


(3) $$\begin{array}{l} D_{i}=\left[I_{i-2}-I_{i-1},I_{i-1}-I_{i},I_{i}-I_{i+1},I_{i+1}-I_{i+2}\right] \end{array}$$

To enhance the precision of motion trend information for action recognition, the proposed method leverages bidirectional information (Li et al., 2023), achieving sufficient capture of motion features and critical visual cues without the need for introducing excessive parameters. Specifically, we denote *I*_*i*−1_ and *I*_*i*+1_ as the adjacent frames within the same segment for the *I*_*i*_ frames from segment *i*. Thus, could obtain the forward and backward temporal differences as:


(4) $$\begin{array}{l} D_{i}^{f}=\left\{I_{i}-I_{i-1}\right\} \end{array}$$

(5) $$\begin{array}{l} D_{i}^{b}=\left\{I_{i}-I_{i+1}\right\} \end{array}$$

Considering that subtracting adjacent frames results in only N-1 temporal difference frames, we prepend and append a zero frame to both the forward temporal difference sequence $D_{i}^{f}$ and the backward temporal difference sequence $D_{i}^{b}$. This adjustment ensures that the summation of forward and backward frame differences is staggered at the zero frame, thereby simplifying the computation of the overall temporal difference. To maintain consistency, we continue to use $D_{i}^{f}$ and $D_{i}^{b}$ to denote these sequences after restoration, and based on these frames, we compute the total temporal difference sequences:


(6) $$\begin{array}{l} D_{i}=D_{i}^{f}+D_{i}^{b} \end{array}$$

Considering that in some adjacent frames, subtle changes in human motion may result in temporal difference frames lacking temporal information, we compute the average of the overall temporal differences to comprehensively capture global temporal information. This approach effectively adjusts and restores the temporal difference sequence to *T* frames:


(7) $$\begin{array}{l} D\left(I_{i}\right)=AVG\left(D_{i-2},D_{i-1},D_{i},D_{i+1},D_{i+2}\right) \end{array}$$

**Figure 3:**
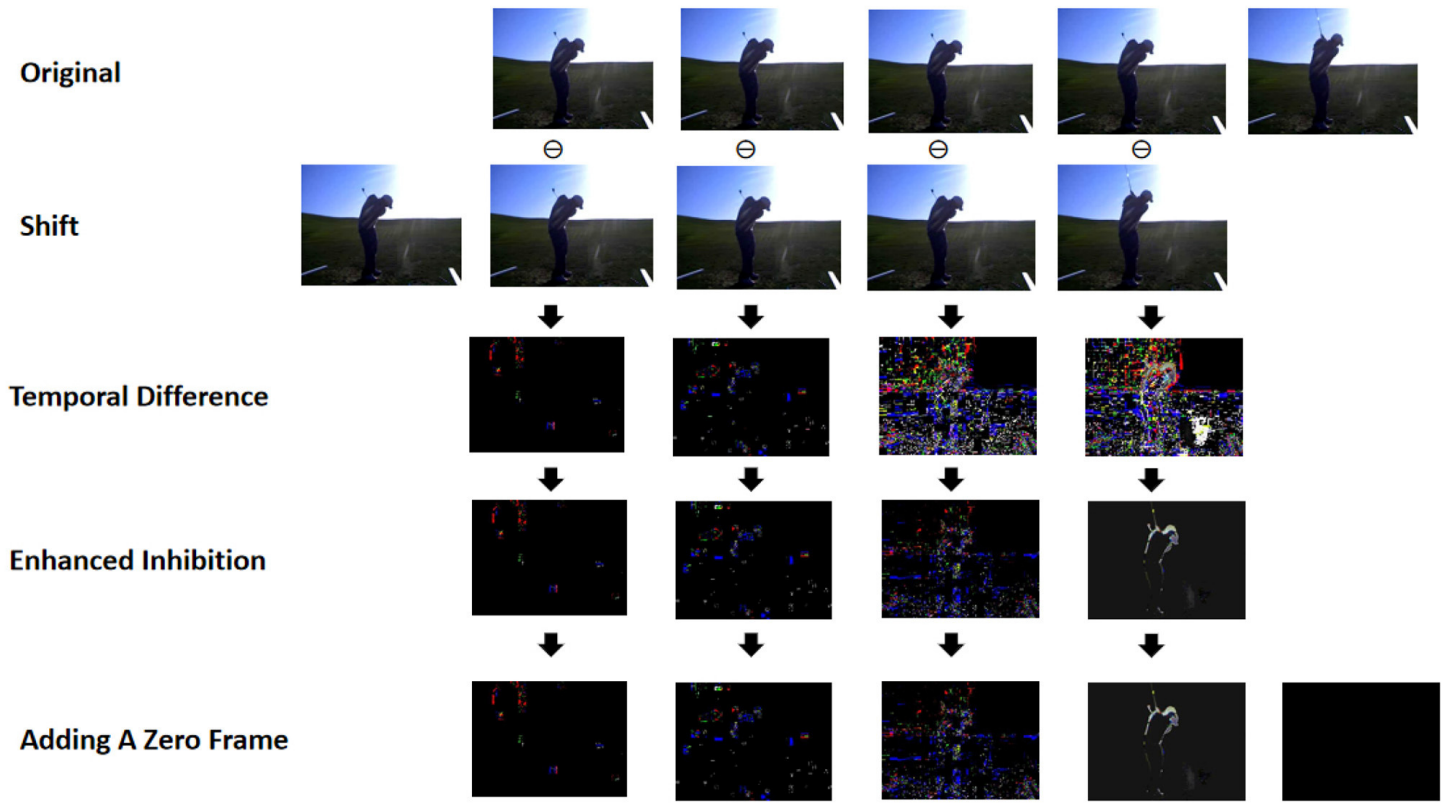
Overview of the temporal difference inhibition studied in the Short-term Motion Information Frame.

Image noise and illumination changes can cause short-term temporal differences that mistakenly register as motion, even when only the target figure itself is actually transforming. To address this, inspired by the motion focus concept from SSTSA (Alfasly et al., 2024), we propose the Temporal Difference Inhibition (TDI) method, as depicted in Figure 3. Here, we introduce a threshold α to the differential frame image, setting pixel values below this threshold to zero, thus minimizing noise-induced discrepancies:


(8) $$D_{\left(T-1,c,h,w\right)}=\{\begin{array}{l} 0,\;if(D\leq \alpha \times k)\\ D+\beta \times k,\;if(D>\alpha \times k) \end{array}$$

Where α is the threshold value and 1≥α≥0, *k* = *I*_*max*_−*I*_*min*_ acting as the scale factor to adjust for the range of pixel intensity values in the input image–i.e., *k* = 255 for non-normalized input *D*. Additionally, β serves as an enhancement multiplier, amplifying the pixels indicative of actual movement, thereby enhancing motion detection while suppressing irrelevant changes.

### 3.3 Long-term motion information

While the SMIF frame-based representation excels in encapsulating local spatio-temporal information, its application is confined to intra-segment video analysis, thereby limiting its scope in probing the extended temporal dimensions of action models. Drawing inspiration from META (Ye et al., 2022) and CLS-Net (Xue et al., 2023), our attempt at LMIM focuses on leveraging inter-segmental data to bolster the representation of prolonged temporal information. This is achieved through a bidirectional multiscale temporal difference module, tailored for long-duration analysis.

**Figure 4:**
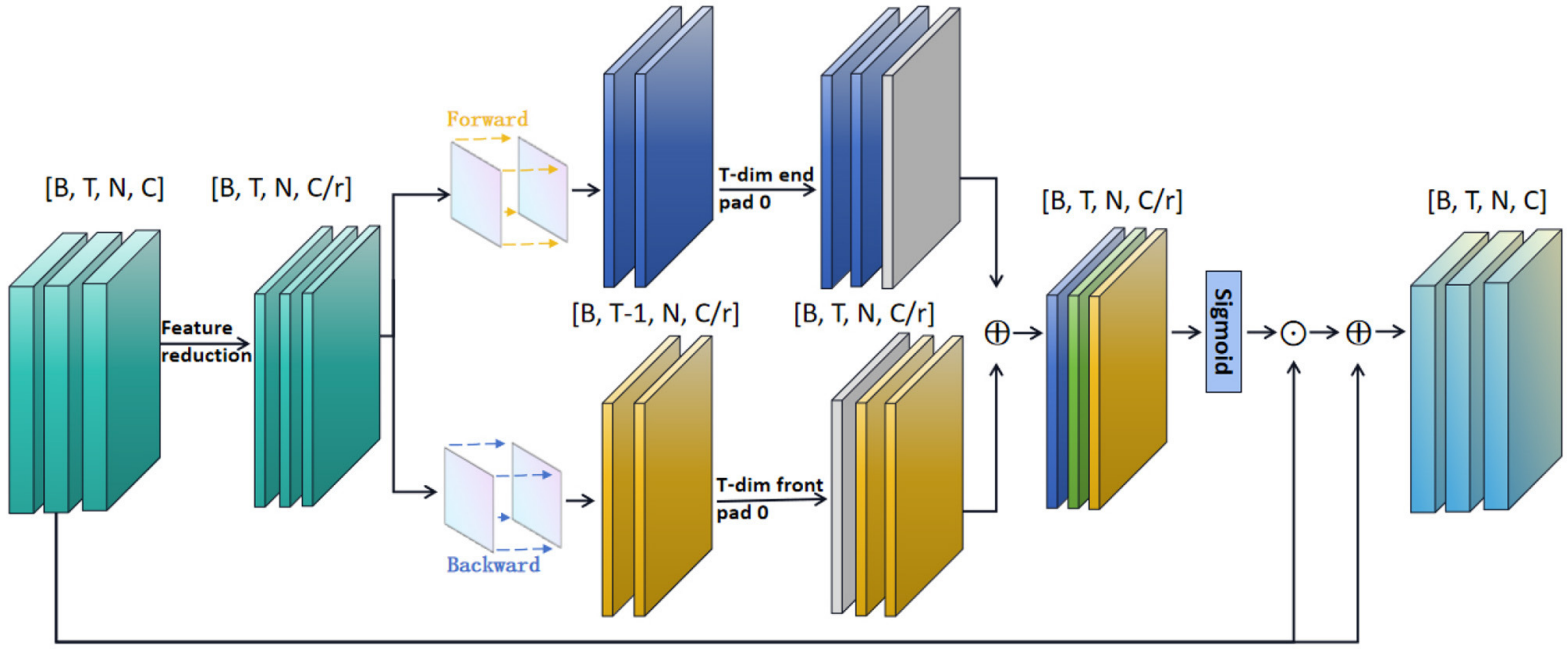
Illustration of the Long-term Motion Information Module. The channel feature dimensions are reduced, and then, after differential processing, a dot product on the channel is done with the original feature. ⊙ denotes the element-wise matrix multiplication. ⊕ denotes the element-wise sum.

Figure 4 illustrates the Long-term Motion Information Module (LMIM), which is integrated into the block *l*. This module is designed to optimize challenges arising from spatial displacements between frames of long-term motion information, while also reducing feature dimensions to enhance computational efficiency. Specifically, the segment features input into the model are represented as *F* with dimensions [*B, T, N, C*]. By employing channel feature averaging, we reduce the feature dimensions of *F* to 1/*r* of the original size, obtaining a new feature representation $\hat{F}$ with dimensions [*B, T, N, C*/*r*]. Subsequently, we calculate the temporal differences across all segments:


(9) $$\begin{array}{l} C_{f}=\hat{F_{i}}-\hat{F_{i-1}} \end{array}$$

(10) $$\begin{array}{l} C_{b}=\hat{F_{i}}-\hat{F_{i+1}} \end{array}$$

After obtaining the bidirectional temporal differences, a method similar to SMIF is employed to convert them back to the original *T* segments. To capture temporally salient features, the Sigmoid function is applied to normalize the learned attention coefficients, generating a temporal attention map. A hyperparameter δ is used to regulate the intensity of the temporal attention map. Based on empirical practices [such as in TDN (Wang et al., 2021)], δ is set to 0.5, ensuring that the attention-enhanced features are primarily driven by the original features:


(11) $$\begin{array}{l} A_{c}=Sigmoid\left(C_{f}+C_{d}\right)-\delta \end{array}$$

Since the temporal attention map can simultaneously capture both spatially and semantically relevant features, it enhances the spatio-temporal representation of the features:


(12) $$\begin{array}{l} F_{i}⊙L\left(F_{i}\right)=F_{i}⊙A_{c} \end{array}$$

where ⊙ is the element-based multiplication, this approach not only integrates the original frame-level representations but also enhances them through the residual connection defined in Equation 2. The strategy of combining multiplication and addition effectively activates key information within the original features, enabling the network to focus on both spatially and semantically relevant features. Simultaneously, it maintains the integrity of background features to a certain extent, thereby achieving accurate action recognition. SMIF and LMIM complement each other by providing information to each other and assisting the model in the extraction of features.

## 4 Experiments

In this section, we present the results of our LS-VIT experiments. First, we outline the datasets assessed and the precise methodologies employed. The following sections are dedicated to exploratory ablation studies focused on LS-VIT's architecture, resulting in a comparative analysis against leading-edge methods.

### 4.1 Datasets and implementation details

#### 4.1.1 Video datasets

We evaluate our model on two datasets, UCF101 (Soomro et al., 2012) and HMDB51 (Kuehne et al., 2013). UCF101 is a collection of 13,320 internet-sourced video clips, categorized into 101 human action classes. Conversely, HMDB51, with its 6,776 video clips sourced from a diverse array of origins including films and internet videos, catalogs 51 varieties of human actions. Kinetics-400 (Carreira and Zisserman, 2017) is a large-scale YouTube video dataset comprising approximately 300,000 video clips across 400 categories. This dataset includes a wide range of activities from daily life, with some categories being highly correlated with specific objects of interaction or contextual scenes.

#### 4.1.2 Training

In this paper, the model is chosen to be based on the VIT implementation, and *T* = 16 frames are extracted from each video. MixUp data augmentation is applied with an alpha value of 0.8, and label smoothing is set to 0.1. By convention (Wang et al., 2018; Feichtenhofer et al., 2018), we adjust the shorter dimension of video frames to 256 pixels during the training phase, and cropping these to a uniform 224 × 224 pixel dimension. The LS-VIT fine-tuning of the model is achieved on the K400 dataset, utilizing the ImageNet (Deng et al., 2009) dataset as a foundational resource. Key training parameters include an initial learning rate of 0.003, a learning rate decay of 0.001, across a total training span of 50 cycles. Experiments are conducted on a GTX 3090 with a memory of 24 GB.

### 4.2 Ablation studies

This paper also describes a comprehensive set of experiments conducted primarily on the HMDB51 dataset, leveraging the VIT-B backbone network (Dosovitskiy et al., 2021)–pre-trained utilizing ImageNet21k and K400 datasets. These experiments, designed with *T* = 16 and *D* = 5 parameters, were adopted to provide clarity through ablation experiments.

#### 4.2.1 Research on the effectiveness of SMIF

The Short-term Motion Information Frame is produced by integrating the short-term frame difference image with the base image with a further process of scaling and weighting after considering the critical nature of traditional image pixel values. As illustrated in Table 1, various base images, when merged with differing magnitudes of frame difference, yield different modes of image representation. We assess how varying scales affect the precision of the resultant image. Notably, in short-term motion information imagery, an enhanced representation is achieved when the frame difference image's proportion surpasses that of the base image. This improvement is attributed to the suppression of temporal discrepancies in the frame difference image, thereby enriching its integration with the base image and more effectively conveying motion information.

**Table 1:** Effect of different scales on SMIF, where *X* denotes the original image and *D* denotes the average frame difference.

**Ratio**	**Top 1**
0.8X+0.2D	74.3%
0.7X+0.3D	74.8%
0.6X+0.4D	75.6%
0.5X+0.5D	75.4%
0.4X+0.6D	**75.7%**
0.3X+0.7D	75.4%
0.2X+0.8D	74.7%

The bold values represent the highest accuracy.

#### 4.2.2 The influence of threshold and enhancement factor on SMIF

By setting a pixel-level change threshold α, static regions are subdued, thus simplifying the task of accentuating dynamic sections. This study indicates that overlooking minor pixel fluctuations allows for a sharper focus on the essence of movement, despite the fact that pixels across successive motion frames exhibit variability in motion intensity. In this context, we employed the LS-VIT model, experimenting with four thresholds: α = 0.02, α = 0.05, α = 0.07, and α = 0.1. The findings, presented in the initial segment of Table 2, demonstrate that a threshold of α = 0.02 outperforms its counterparts, effectively considering up to 98% of pixel activity as significant motion. Thereafter, we explore the effect of the amplification factor, β, which scales the intensity of motion in active regions by a factor of β, while static regions are rendered inert. Experimenting with values β = 0.02, β = 0.05, β = 0.07, and β = 0.1, and the second part of Table 2 indicates that LS-VIT performs best with β = 0.07.

**Table 2:** Effect of different thresholds *α* as well as enhancement multiplicity *β* on SMIF.

**α**	**β**	**Top 1**
0	0	75.7%
0.02	0	**75.9%**
0.05	0	75.6%
0.07	0	75.8%
0.1	0	75.2%
0.02	0.02	76.1%
0.02	0.05	76.2%
0.02	0.07	**76.5%**
0.02	0.1	75.8%

The bold values represent the highest accuracy.

#### 4.2.3 Research on the impact of different channel reduction rates in LMIM on LS-VIT

In Section 3 Methodology, the study evaluates the rationale for proposing LMIM, which aims at reducing the number of channel features to better align motion information. Specifically, for the VIT during the Attention phase, the number of channel features stands at *C* = 768. To streamline the computational process and adjust tensor shapes more efficiently, various reduction quantities were experimented with, including *r* = 2, *r* = 3, *r* = 4, *r* = 6, and *r* = 8. According to Table 3, it is observed that reducing the image channels to 1/6 of their original number (*r* = 6) offers a more effective solution to the image alignment challenge, demonstrating optimal performance of LMIM in this context.

**Table 3:** Effect of different channel feature reduction number ratios *r* on the LMIM.

**Reduce quantity**	**Top 1**
1	71.7%
2	71.9%
3	71.4%
4	71.5%
6	**72.0%**
8	70.6%

The bold values represent the highest accuracy.

#### 4.2.4 Effect of SMIF vs. LMIM on LS-VIT

SMIF and LMIM are integrated at different points in the model, each contributing uniquely to the processing of long-term and short-term motion information. This complementary action is assessed by comparing the optimal performances of both SMIF and LMIM in enhancing LS-VIT identification, as outlined in Table 4. Individually, SMIF and LMIM enhance VIT identification capabilities, with SMIF demonstrating a slight edge over LMIM. However, combining them does not significantly amplify the results. The reason for this phenomenon is that the SMIF encompasses not only the spatial information essential for action representation but also a portion of short-term motion information. In the context of the Vision Transformer (VIT), the focus is predominantly on the SMIF. As a result, when both the SMIF and LMIM are employed concurrently, the contribution of the LMIM becomes less pronounced. This observation led to a further study, particularly focusing on LMIM's channel reduction effect on LS-VIT. The findings, illustrated in Table 5, indicate that the combination of SMIF and LMIM is most effective at a reduction ratio of *r* = 1, suggesting that for extracting short-term motion information, LS-VIT benefits from a minimal reduction in channels. At this rate, the module utilizes uncompressed features for temporal differences, this minimal reduction avoids overly compressing the channels, thus adequately capturing long-term motion information without compromising the model's effectiveness.

**Table 4:** Impact of SMIF and LMIM on the model; √ indicates that the module was used; - indicates that it was not used.

**SMIF**	**LMIM**	**Top 1**
-	-	69.1%
-	√	72.0%
√	-	76.5%
√	√	76.9%

**Table 5:** Impact of SMIF with different LMIM reduction ratios.

**SMIF_Ration**	**LMIM_Ration**	**Top 1**
0.4X+0.6D	1	**77.0%**
0.4X+0.6D	2	76.7%
0.4X+0.6D	3	76.3%
0.4X+0.6D	4	76.4%
0.4X+0.6D	6	76.8%
0.4X+0.6D	8	76.0%

The bold values represent the highest accuracy.

### 4.3 Comparison with the state of the art

In Table 6, we adhered to the universally accepted evaluation protocol for this dataset to maintain an equitable benchmark. The findings from these tests indicate that our model surpasses both TSM and TDN in performance metrics, especially significant when the input consists of 16 frames. Considering the action categories in the HMDB51 dataset have a high correlation to motion data, the capacity for temporal analysis is crucial. Our model demonstrates proficiency in capturing and leveraging temporal data, leading to a enhancement in recognition capabilities.

**Table 6:** Comparison with state-of-the-art methods on HMDB51.

**Model**	**Pretrain**	**FLOPS**	**Params**	**Top 1**
TSM (Lin et al., 2019)	K400	65G	24.3M	73.2%
STM (Jiang et al., 2019)	K400	66.5G	24M	73.3%
TEA (Li et al., 2020)	K400	70G	24.3M	72.2%
TDN (Wang et al., 2021)	K400	72G	24.8M	76.3%
TCM (Liu Y. et al., 2022)	K400	105G	49M	**77.5%**
Timesformer (Bertasius et al., 2021)	K400	196G	122.2M	72.7%
Video-Swin (Liu Z. et al., 2022)	K400	282G	88.1M	72.2%
Vidtr (Zhang Y. et al., 2021)	K400	179G	61M	74.4%
MSVL (Chen et al., 2024)	ImageNet	158G	27.3M	72.9%
LS-VIT (Ours)	K400	134.9G	85.69M	77.0%

The bold values represent the highest accuracy.

Further comparative analysis was carried out against the latest leading methods on the UCF-101 dataset, with detailed results presented in Table 7. Echoing the patterns observed with the HMDB51 dataset, our LS-VIT model delivers performance on par with other leading models under the condition of utilizing 16 frames as input. It is important to highlight that the UCF-101 dataset's comparatively limited scope allows most models to reach near-peak accuracy levels, particularly when pre-trained with “ImageNet + K400.” For instance, TDN's accuracy peaks at 97.4%. Against this backdrop of high baseline accuracies, enhancing model performance poses a significant challenge. Nonetheless, the comparative data emphasizes that our LS-VIT model manages to closely match, if not rival, the performance of the current SOTA models. This proves the robustness' of LS-VIT in its competitive edge in the field.

**Table 7:** Comparison with state-of-the-art methods on UCF101.

**Model**	**Pretrain**	**FLOPS**	**Params**	**Top 1**
TSM (Lin et al., 2019)	K400	65G	24.3M	96.0%
STM (Jiang et al., 2019)	K400	66.5G	24M	96.2%
TEA (Li et al., 2020)	K400	70G	24.3M	96.9%
TDN (Wang et al., 2021)	K400	72G	24.8M	97.4%
TCM (Liu Y. et al., 2022)	K400	105G	49M	97.2%
Timesformer (Bertasius et al., 2021)	K400	196G	122.2M	94.7%
Video-Swin (Liu Z. et al., 2022)	K400	282G	88.1M	**97.6%**
Vidtr (Zhang Y. et al., 2021)	K400	179G	61M	96.6%
MSVL (Chen et al., 2024)	ImageNet	158G	27.3M	97.6%
LS-VIT(Ours)	K400	134.9G	85.69M	97.1%

The bold values represent the highest accuracy.

In Table 8, we present a detailed report of the experimental results on the Kinetics400 dataset, which focuses on scene-based action recognition, and compare our findings comprehensively with current state-of-the-art techniques. The data clearly show that our proposed LS-VIT model achieves a top-1 accuracy of 74.9%. This performance significantly surpasses most (2+1D) CNN-based methods such as TSM and TEA, and is comparable to the TDN model. Compared to Transformer-based methods like Timesformer and Vidtr, LS-VIT also excels in accuracy performance and significantly reduces FLOPS, further highlighting its advantages in both performance and computational efficiency.

**Table 8:** Comparison with state-of-the-art methods on Kinetics400.

**Model**	**Pretrain**	**FLOPS**	**Params**	**Top 1**
TSM (Lin et al., 2019)	ImageNet	65G	24.3M	72.6%
STM (Jiang et al., 2019)	ImageNet	66.5G	24M	73.7%
TEA (Li et al., 2020)	ImageNet	70G	24.3M	74.0%
TDN (Wang et al., 2021)	ImageNet	72G	24.8M	75.5%
Timesformer-L (Bertasius et al., 2021)	ImageNet	2380G	121.4M	80.7%
Video-Swin (Liu Z. et al., 2022)	ImageNet	282G	88.1M	**82.7%**
Vidtr (Zhang Y. et al., 2021)	ImageNet	179G	61M	78.6%
LS-VIT (Ours)	ImageNet	134.9G	85.69M	74.9%

The bold values represent the highest accuracy.

While methods like Timesformer and Videoswin demonstrate strong performance in video processing, their efficiency is limited by a heavy reliance on three-dimensional convolutions, which undoubtedly increases computational costs. In contrast, LS-VIT not only excels in accuracy but also significantly reduces FLOPS, further highlighting its advantages in both performance and computational efficiency. Although TEA and TSM are known for their computational efficiency, they may struggle to capture subtle spatiotemporal details. In such cases, LS-VIT, through its innovative bidirectional motion capture strategy, provides superior performance. By leveraging the synergistic functionality of the SMIF and LMIM modules, LS-VIT accurately captures and analyzes motion information without imposing additional computational burdens.

**Figure 5:**
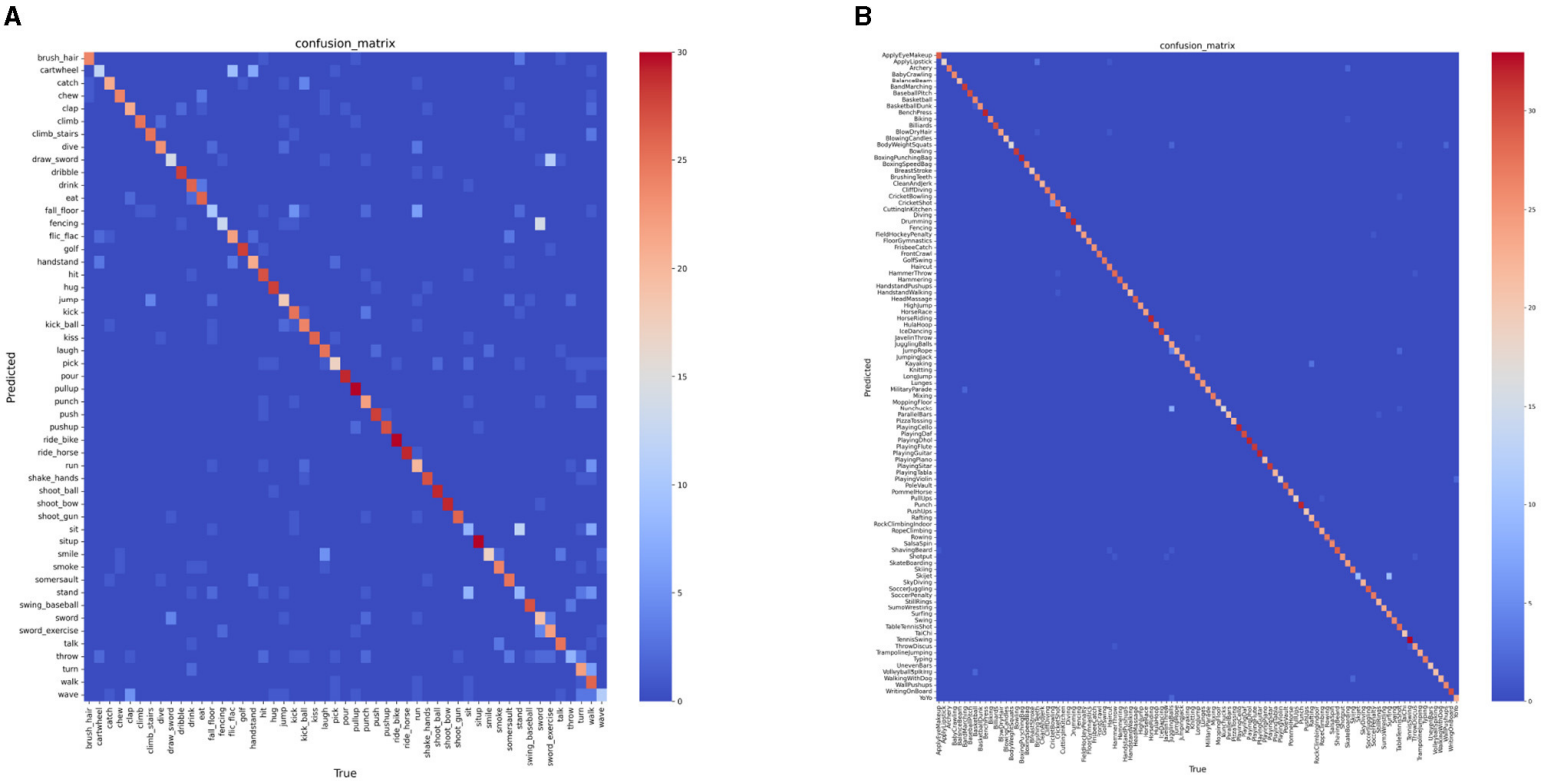
Confusion matrix of LS-VIT on the HMDB51 **(A)** and UCF101 **(B)**.

To more intuitively evaluate the performance of LS-VIT on the HMDB51 and UCF101 datasets, we have visualized the confusion matrices and presented them in Figure 5. In this figure, the intensity of the red color in the diagonal elements represents the prediction accuracy of the model for each class; the deeper the red, the higher the prediction accuracy for that category.

## 5 Conclusions and discussions

To address the challenge of detecting actions within videos, our study introduces the LS-VIT network, which analyzes both long- and short-term motion differences. This network is specifically designed to model temporal information in a bidirectional manner, effectively capturing motion trends from both forward and backward perspectives. This approach aims to overcome common hurdles such as high computational demand and inefficient use of temporal data that plague current methodologies.

We conducted rigorous testing of the LS-VIT network across three widely recognized benchmark datasets, allowing for a thorough analysis of each component. The results, obtained under consistent experimental conditions, indicate that the LS-VIT network demonstrates effective utilization of temporal information, offering improvements over its predecessors in the field of action recognition.

Our model performs well with actions involving complex motion patterns, such as dance or martial arts. However, it may struggle to capture sufficient motion information for actions with smaller ranges or slower speeds, such as subtle facial expressions or finger movements, which can affect recognition accuracy. Additionally, the model is primarily optimized for single-person action recognition, and challenges increase in multi-person interaction scenarios due to the need to account for dynamic interactions and occlusion issues.

Future research will focus on testing the model on more diverse datasets, including multi-person interaction scenarios and domain-specific actions, to evaluate and improve its generalization ability. Subsequent work may extend this method to other areas, such as action prediction and real-time action detection. We also plan to emphasize improving training speed and reducing inference time while exploring applications of this approach in action prediction.

## Data Availability

Publicly available datasets were analyzed in this study. This data can be found here: https://www.crcv.ucf.edu/data/UCF101.php, https://serre-lab.clps.brown.edu/resource/hmdb-a-large-human-motion-database/, and https://deepmind.com/research/open-source/kinetics.
